# Malignant infantile osteopetrosis in a 3-year-old Yemeni child: a case report

**DOI:** 10.11604/pamj.2022.43.30.36827

**Published:** 2022-09-19

**Authors:** Saeed Thabet, Mohammed Almajeedi, Maged Mohammed, Faisal Ahmed

**Affiliations:** 1 Hematology and Internal Medicine Department, Faculty of Medicine, Taiz University of Medical Sciences, Taiz, Yemen,; 2 Department of Internal Medicine, Saeed Thabet Nasher Hospital, Taiz, Yemen,; 3 Department of Internal Medicine, Faculty of Medicine, Taiz University of Medical Sciences, Taiz, Yemen,; 4 Urology Research Center, Al-Thora General Hospital, Department of Urology, School of Medicine, Ibb University of Medical Sciences, Ibb, Yemen

**Keywords:** Infantile malignant osteopetrosis, hepatosplenomegaly, case report

## Abstract

Infantile malignant osteopetrosis (IMOP) is a rare bone resorptive disorder with an autosomal recessive inheritance pattern. It is characterized by increased bone density due to osteoclastic failure in differentiation or function. The clinical manifestations of IMOP start at birth or infancy with varied rings according to the type and degree of osteopetrosis. We presented a 3-year-old female patient referred to us due to chronic anaemia six months ago. The physical examination revealed hepatosplenomegaly, axial hypotonia, and visual impairment. Blood investigation revealed pancytopenia and hypocalcemia. Radiologic studies revealed a generalized increase in bone density, abnormal metaphyseal remodelling, and rain atrophy. The bone marrow aspiration (BMA) shows dry tap and hypocellularity of all cell lines. IMOP was diagnosed depending on clinical, radiologic, and BMA results. In conclusion, IMOP is relatively uncommon. Accurate diagnosis should be made through clinical, BMA, and radiologic investigations, especially in a resource-limited setting, as performed in our case.

## Introduction

Infantile malignant osteopetrosis (IMOP) is a rare bone resorptive disorder with an autosomal recessive inheritance pattern and poor outcome [1]. IMOP incidence rate is 1 in 250,000 newborns [2]. It is characterized by increased bone density due to osteoclastic failure in differentiation or function, which prevents normal bone resorption and remodelling [1]. IMOP includes various clinical features, including hematologic and neurological manifestations, and is usually diagnosed based on clinical, radiologic, and BMA evaluations [1,3]. There are limited reports in the literature regarding IMOP, especially in a resource-limited setting [1-4]. Here, we report a case of IMOP with brain atrophy in a 3-year-old Yemeni child.

## Patient and observation

**Patient information:** a 3-year-old child female was referred to the oncology department due to chronic anaemia and hepatosplenomegaly six months ago. She was born at term with normal vaginal delivery after an uncomplicated pregnancy. Parents reported delayed developmental milestones, growth retardation, delayed teeth eruption, compared to a child at the same age, and poor visual contact for three months of birth. The parents were non-consanguineous, and no anomalies or congenital disorders were recorded in her family.

**Figure 1:**
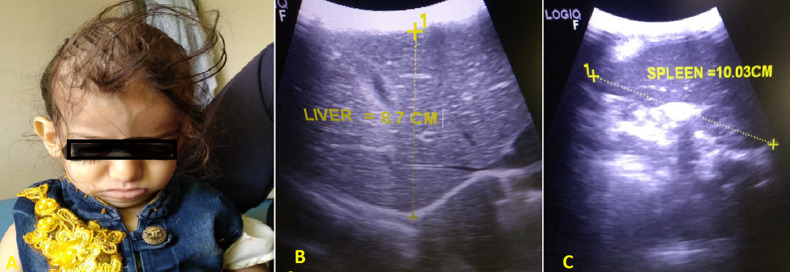
A) frontal bossing; ultrasonography image showing; B) hepatomegaly; C) splenomegaly

**Clinical Findings**: the patient weight: 7 kg, height: 52m, and body mass index (BMI): 25.9 kg/m^2^. Head and neck examination revealed frontal bossing depressed nasal bridge (Figure 1 A). The ophthalmic examination reveals convergent squinting with loss of visual fixation and pursuit, while the fundoscopic examination reveals bilateral optic atrophy. Examination of the lung and cardiovascular system was within normal limits. Abdominal examination showed a distended abdomen with hepatosplenomegaly. External and internal genitalia were revealed to be normal. Lower limbs examination shows knee valgus and hypotonia.

**Diagnostic assessment:** the white blood cells: 7.6 ×10^3^/ml, hemoglobin: 5 g/L (severe anaemia), platelets: 26×10^3^/ml (thrombocytopenia), vitamin D: 25.52 ng/mL, calcium: 7.8 mg/dL (hypocalcemia), and elevated parathyroid hormone (PTH): 152 pg/mL. The other blood investigation tests were within normal limits, including alkaline phosphatase, liver function tests, renal function tests, and immunoglobulin levels (IgG, IgA, and IgM) were within normal ranges. The serology tests of toxoplasma, hepatitis, cytomegalic, and rubella viruses were negative.

**Figure 2:**
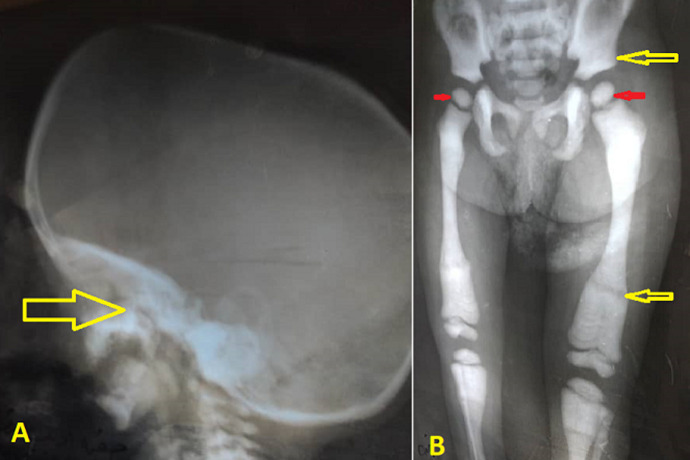
A) lateral skull radiograph showing increased thickness of skull base (arrow); B) increased bone density with little differentiation between cortex and medulla in long bone and pelvis (yellow arrows), metaphyseal modeling defects, and characteristic lucent bands (red arrow)

**Figure 3:**
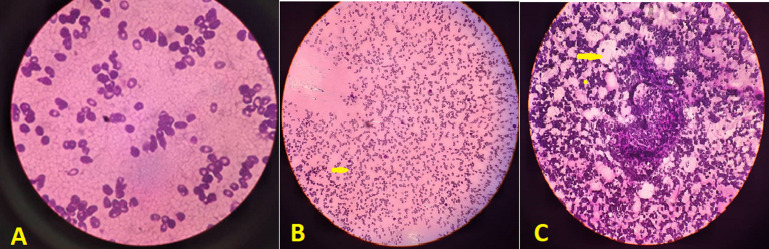
bone marrow aspiration showing A) dry tap and hypocellularity of all cell lines; B) myelosclerosis with no cellular fragments (arrow); C) no hematopoietic cells with increased fat cells (arrow)

**Figure 4:**
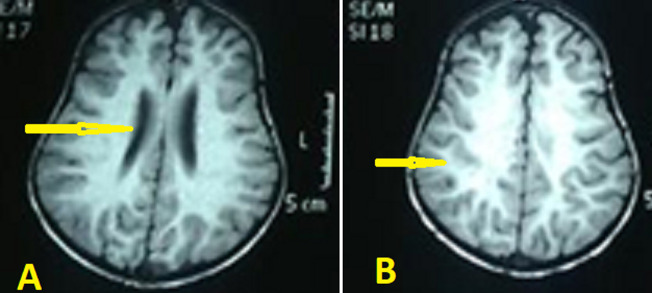
brain magnetic resonance imaging showing A) mild dilated third ventricle (arrow); B) prominent cortical sulci and sylvan fissures (arrow)

Abdominal ultrasonography (US) revealed hepatosplenomegaly (liver span: 8.7cm, spleen span: 10.3cm), and other abdominal organs were within a normal appearance (Figure 1 B, C). Imaging radiologic plain X-ray studies showed an increase in bone density and thickness, particularly in the skull base (Figure 2 A), and bone modeling affected the metaphysis of long bones (Figure 2 B). Bone marrow aspiration (BMA) shows dry tap and hypocellularity of all cell lines (Figure 3). Brain magnetic resonance imaging (MRI) shows mild dilated cerebral ventricles with no midline shift or deformities. Prominent cortical sulci, sylvan fissures, extracerebral cerebrospinal fluid (CSF) space, and anterior interhemispheric fissure. All these findings were suggestive of brain atrophy (Figure 4). IMOP was diagnosed depending on clinical, radiologic, and BMA results. The patient family was advised to perform genetic testing, which was not performed due to the high cost and lack of availability in our city.

**Therapeutic interventions:** antibiotic therapy, blood transfusion, and calcium and vitamin D supplementation were all used to treat the patient's symptoms. The patient family was advised to perform hematopoietic stem cell transplantation (HSCT), which was not performed due to the high cost and lack of availability in our city.

**Follow-up and outcome:** within six months of follow-up, the patient was still alive and received supportive management with regular pediatric follow-up. The prenatal screening was recommended for subsequent pregnancies.

**Patient perspective:** during treatment, the patient´s families were satisfied with the level of care provided to her. The patient´s families understood the poor prognosis of the patient disease.

**Informed consent:** the consent was obtained from the patient's family for publication of this case, including figures.

## Discussion

Osteopetrosis is a diverse group of diseases characterized by increased bone density on radiographs due to osteoclastic failure in differentiation or function. There are four subtypes of osteopetrosis: IMOP, benign or adult osteopetrosis, intermediate osteopetrosis, and carbon anhydrase type II deficiency [2]. Mutations mainly cause IMOP in the CLCN7 (chloride voltage-gated channel 7) and TCIRG1 (T cell immune regulator 1) gene, with an incidence rate of 1 in 250,000 newborns. Milder forms of autosomal recessive osteopetrosis are caused by mutations in the PLEKHM1 (Pleckstrin homology domain-containing family M member 1) gene [5]. Up to two-thirds of these patients die before age six, and many recurrent infections, especially pneumonia or osteomyelitis, complicated by septicemia, are the leading causes of death [3].

IMOP is a rare type with an autosomal recessive inheritance pattern that typically begins prenatally, manifests at birth or in infancy, and is associated with greater severity than other forms [1]. Our patient has had the symptoms since she was four months old. Phenotypic characteristics such as macrocephaly and frontal bossing due to increased bone mass, defects in tooth eruption, short stature, and a propensity for fractures and osteomyelitis are common in these patients. The hematologic manifestations are anaemia, thrombocytopenia, risen infection susceptibility, and secondary development of extramedullary hematopoiesis sites such as the liver and spleen result from abnormal bone development [1,6,7]. Except for bony fractures and osteomyelitis, our reported case exhibited all these characteristics.

Neurological manifestations of this syndrome result in obstruction of the foramina through which the cranial nerves, spinal cord, and major blood vessels pass through the skull, causing impaired vision, deafness, facial paresis, and hydrocephaly. Some patients with autosomal recessive osteopetrosis variants (neuropathic ARO) exhibit signs of principal neurodegeneration, such as seizures with normal calcium levels, growth retardation, hypotonia, retinal atrophy, and neural hearing deafness, in addition to these compressive occurrences [7]. Children with IMOP likely develop Rickets and hypocalcemia, leading to tetanic seizures and secondary hyperparathyroidism [3,5]. Except for seizure, our patient had hearing deafness, visual impairment, hypocalcemia, and rickets.

Radiographic findings of IMOP include a significant bone density increase and malfunctioning metaphyseal remodelling in long bones. Brain MRI findings of IMOP include severely delayed myelination and diffuse progressive cortical and subcortical atrophy, as seen in our patient [1,3]. BMA biopsy can differentiate between osteoclast-poor and osteoclast-rich variants of IMOP; our patient's BMA biopsy revealed infrequent osteoclasts [1]. Karyotyping can ensure the diagnosis and distinguish between osteopetrosis subtypes, offering extra information about prediction, likely response to treatment, and recurrence risks [2]. In our case, the patient family was advised to perform genetic testing, which was not performed due to the high cost and lack of availability in our city.

IMOP is primary bone sclerosis and must be distinguished from numerous conditions in which bone sclerosis can occur as a secondary phenomenon [8]. Pseudohypoparathyroidism, pyknodysostosis, hypoparathyroidism, organic intoxication (e.g., lead, fluoride, and beryllium), and malignancies (myeloproliferative diseases and leukemia) are some alternative diagnoses to consider [1,8]. In our case, the final diagnosis was made depending on clinical, radiographic, and BMA findings.

Treating patients with osteopetrosis necessitates a comprehensive approach to the underlying clinical issues, including hematological and biochemical abnormalities, recurrent infections, bone consequences, and neurodevelopmental morbidities [3,9]. HSCT is currently the only cure option for IMOP; it should be performed as soon as possible to avoid irrevocable neurologic and cognitive deficits [10]. The high morbidity and mortality associated with HSCT are reserved only for the most severe cases of osteopetrosis. Patients who received allogeneic donor stem cell transplants had positive outcomes. Moreover, non-allogenic HSCT may be an option for treating IMOP, as it demonstrated a high survival rate and restoration of hematopoiesis in haploid transplant patients [11]. Because of the non-availability of HSCT in our city, treatment was largely supportive and focused on providing surveillance and symptomatic management of complications such as antibiotic therapy, calcium and vitamin D supplementation, and nutritional measures [1].

Genetic screening is critical. Prenatal identification of IMOP in families using radiographic images may be feasible, allowing HSCT to improve neurological consequences before three months [12]. However, the challenge of getting definitive results from the prenatal sonographic assessment of the fetus makes prenatal molecular diagnosis highly attractive [6]. Similarly, we recommended prenatal screening for subsequent pregnancies.

## Conclusion

IMOP is a rare differential diagnosis of hepatosplenomegaly. An accurate diagnosis should be obtained through clinical signs, radiologic investigations, and gene sequencing. Prompt HSCT is critical for effective management and preventing the disease progression before irreversible neurological subsequences.
